# Generational analysis of trends in unprotected sex in France among men who have sex with men: The major role of context-driven evolving patterns

**DOI:** 10.1371/journal.pone.0171493

**Published:** 2017-02-07

**Authors:** Nicolas Méthy, Laurence Meyer, Nathalie Bajos, Annie Velter

**Affiliations:** 1 CESP, Univ. Paris-Sud, UVSQ, INSERM, Université Paris-Saclay, Villejuif, France; 2 Santé Publique France, Saint Maurice, France; British Columbia Centre for Excellence in HIV/AIDS, CANADA

## Abstract

**Objective:**

Using a generational approach, this study analyses how unprotected anal intercourse has evolved since 1991 in France across different generations of men who have sex with men (MSM) whose sexual lives began at different periods in the history of the HIV epidemic.

**Design:**

Data were collected from 18–59 year-old respondents to the French Gay Press surveys *Enquêtes Presse Gay*, conducted repeatedly between 1991 and 2011 (N = 32,196) using self-administered questionnaires distributed in gay magazines and over the internet.

**Methods:**

Trends in unprotected anal intercourse (i.e. condomless anal sex) with casual partners of unknown or different HIV serostatus (hereafter “UAId” in this manuscript) were studied. Responses were analysed according to year and then reorganised for age-cohort analyses by generation, based on the year respondents turned 18.

**Results:**

UAId rates fell from 1991 to 1997, and then rose from 13.4% in 1997 to 25.5% in 2011 among seronegative respondents, and from 24.8% to 63.3%, respectively, among seropositive respondents. Both in seropositive and seronegative respondents, UAId increased over time for all generations, indicative of a strong period effect.

**Conclusion:**

Analyses of data from several generations of MSM who started their sexual lives at different time points in the HIV epidemic, revealed very similar trends in UAId between generations, among both seropositive and seronegative respondents. This strong period effect suggests that sexual behaviours in MSM are influenced more by contextual than generational factors. The fact that prevention practices are simultaneously observed in different generations and that there are most likely underlying prevention norms among MSM, suggests that PrEP could become widely accepted by all generations of MSM exposed to the risk of HIV.

## Introduction

Men who have sex with men (MSM) continue to be a population strongly affected by HIV in both high- and low-resource countries [1]. International comparisons have shown the fundamental role of political, structural and cultural factors in the propagation and management of this disease’s epidemic [2]. In high-resource countries, the epidemic among MSM has been widely documented since the first cases of AIDS, and analyses of the trends in epidemiological and behavioural indicators have enabled researchers to study the health status and prevention practices of MSM over time. Despite large community and government investment in prevention and access to care in these countries, the epidemic is still very present in this population [3].

Repeated cross-sectional studies in this population show that MSM prevention practices have changed profoundly since the epidemic began, in France [4,5] as elsewhere [6,7]. The frequency of condom use is currently insufficient to reduce the rate of new infections [8]. Some MSM use alternative strategies to condoms, such as serosorting and seropositioning, which require knowledge of both partners' serostatus. However, these often fail to provide adequate protection [9–12]. Today, the core of prevention involves a combination of screening and either antiretroviral medication (ARV) for the medical management of seropositive individuals (Test and Treat, Treatment as Prevention) [13] or prophylaxis for the most highly exposed seronegative MSM (PrEP) [14,15].

Progress in medicine and changes in public policy have therefore redefined the context of prevention throughout the history of the epidemic. Accordingly, the present epidemiological situation is the result of the constant adaptation of practices by MSM to ever-changing risks. Different prevention strategies, including new biomedical tools, testify to the diversification of prevention practices [16]. A comprehensive analysis of the trends in condom use and the dissemination of other prevention practices over the last 20 years—in terms of their arrival and the extent of their use—would help us to understand the dynamics of this population’s prevention practices today, and perhaps contribute in predicting future trends.

A generational effect on prevention practices has sometimes been hypothesised [17–20]. The underlying hypothesis is that successive generations of MSM have experienced different epidemiological and social contexts during their lifetimes, contexts that have contributed to shaping their specific prevention trajectories over time. Accordingly, the oldest generations, whose sexual lives started long before the AIDS epidemic, may have found it difficult to change their sexual behaviours, use condoms and go for regular screening. Subsequent generations in the 1980s and 1990s were the most affected by AIDS-related deaths and prevention campaigns, and their direct personal and collective experience of the epidemic’s dark years might have left them with a specific relationship with AIDS and prevention, including systematic condom use, as well as its counterpart, condom fatigue. The youngest generations, whose sexual debut occurred in the much more favourable medical context of the 2000s, thanks to the widespread use of combined ARV, may be less well informed about HIV and prevention [21] and therefore more exposed to the risk of infection.

In France, the number of new diagnoses in MSM has continued to rise since 2003 [22], particularly among those under 25 years of age (+157% between 2003 and 2013) [23]. Furthermore the estimated annual HIV incidence rate among MSM was stable and high between 2003 and 2012, approximately 1% per year [24,25].

Collection of data on the prevention practices of MSM in France started in the first years of the epidemic through a unique set of nationwide surveys: the Gay Press surveys (*Enquêtes Presse Gay*) [4,5,26], a series of questionnaires distributed through the gay press and over the internet, repeated periodically between 1985 and 2011. They revealed both an increase in unprotected anal intercourse after 1997 –irrespective of partner type (stable or casual) and HIV serostatus [26]—and poor uptake of alternative risk reduction strategies (serosorting, seropositioning) [27,28]. In addition, the data from these surveys helped us to implement a generational approach retracing sexual trajectories using an age-cohort analysis with graphical representations and multivariate logistic regressions [29]. The results from this approach showed that sexual practices (i.e. masturbation, oral sex, anal intercourse) have evolved over time across age groups and generations, suggesting that widespread community norms exist for these practices and that sexual itineraries of MSM are impacted by current social and epidemiological contexts [29]. The aim of the present study was to use a generational-type analysis to study whether certain high-risk sexual practices, such as unprotected (i.e. condomless) anal sex with casual partners (UAId), followed the same pattern as other previously studied sexual practices [29].

## Materials and methods

### Study population

Our work analyses the Gay Press surveys, a series of socio-behavioural surveys of MSM repeated from 1985 to 2011 (annually from 1985 to 1993, then in 1995, 1997, 2000, 2004 and 2011). These self-administered questionnaires were published in one gay magazine (“Gai Pied Hebdo”) with a large nationwide readership from 1985 to 1992. From 1993 onwards they were published in several other gay magazines to build up a variety of participant profiles similar to those created with Gai Pied Hebdo (6 gay magazines in 1993, 10 in 1995, 9 in 1997, 20 in 2000, 16 in 2004, and one in 2011). In 2004 and 2011, an electronic version of the questionnaire was available on the internet, accessible via banner ads on various information and gay meeting websites (10 sites in 2004 and >60 sites in 2011). The number of participants ranged from approximately 1000 (in 1985 and 1992) to over 10 000 (in 2011). The internet accounted for 23% of the questionnaires analysed in 2004 and 90% in 2011. Questionnaire items collected data on respondents’ socio-demographic profiles, social lives, sex lives (practices and prevention methods) and health.

All questionnaires were completed anonymously. The French data protection committee (*Commission nationale de l'informatique et des libertés*) approved the surveys, data collection and data storage.

The analyses in this study are based on the surveys conducted from 1991 to 2011, except for 1992 (as its variables were not comparable with those of the other surveys). Our analyses are limited to respondents aged 18–59 years.

### Variables for analysis

Risk-taking was defined as reporting at least one episode of unprotected anal intercourse in the 12 months before the survey with a casual partner of unknown or different HIV serostatus (UAId). This indicator was calculated among all respondents who reported having practiced anal intercourse (active and/or passive) at least once in the previous 12 months with any casual partner

Analyses were stratified according to respondents' self-reported HIV serostatus (untested, seronegative, or seropositive). From 2000 onwards, respondents were asked to record their most recent viral load (detectable or not).

### Statistical analysis

The first analysis examined the trends in the percentage of men reporting UAId in the 12 months (by calendar time) before each survey, stratified by serostatus.

We then constructed cohorts (generations) using the starting point of sexual life, from data collected over 20 years, for a longitudinal analysis of these trends. Each generation covered a four-year range starting from the year when respondents turned 18 (the median age of first sexual relationships with a man in these surveys) [29]. As previously described, this allowed to compare trends in UAId by age for different generations of MSM. The trends of the percentage of MSM reporting UAId were studied separately for seronegative and seropositive MSM according to age. We show graphic representations of the longitudinal data only for the era following the widespread use of ARV therapy (1997–2011 surveys) to optimise the legibility of the comparisons.

Respondents' ages in each generation for each survey were described by medians and interquartile ranges (Q1-Q3), and mean ages in each sample were compared by analysis of variance. The qualitative variables were described in percentages, and their distributions compared with the Pearson's Chi-2 test. We used Stata 13 software for data management and statistical analyses.

## Results

### Description of samples (Table 1)

**Table 1:** Social and demographic characteristics, HIV status and UAI in the previous 12 months with casual partners, among respondents to the Gay Press surveys.

	1991 (N = 1757)		1993 (N = 3125)		1995 (N = 2545)		1997 (N = 3205)		2000 (N = 4550)		2004 (N = 5694)		2011 (N = 10 525)	
	n	%	n	%	n	%	n	%	n	%	n	%	n	%
**Age**, years														
Median (Q1-Q3)	32 (27–39)		31 (26–37)		30 (25–35)		31 (26–37)		33 (28–39)		35 (29–42)		35 (26–44)	
**Educational level**														
< High school diploma	431	24.6	727	23.3	501	19.8	631	19.7	727	16.1	1001	17.7	1633	15.7
High school diploma / Bachelor’s degree	666	38.1	1183	38.0	976	38.5	1241	38.8	1669	37.0	2222	39.2	3853	37.0
Master’s Degree/PhD	652	37.3	1205	38.7	1059	41.8	1327	41.5	2119	46.9	2444	43.1	4926	47.3
**Place of residence**														
City < = 100 000 inhab.	621	36.8	762	24.5	708	28.0	867	27.7	1248	27.9	1842	32.9	3857	36.7
City > 100 000 inhab.	465	27.5	725	23.3	626	24.8	878	28.0	1315	29.3	1839	32.8	3229	30.7
Paris area	602	35.7	1618	52.1	1191	47.2	1390	44.3	1918	42.8	1923	34.3	3424	32.6
**HIV serostatus**														
Untested	349	20.2	538	18.1	290	11.6	450	14.2	569	12.8	763	14.0	1366	13.0
Seronegative	1130	65.3	2032	68.3	1860	74.5	2357	74.4	3309	74.3	4103	75.1	7570	72.1
Seropositive	251	14.5	405	13.6	347	13.9	363	11.5	576	12.9	599	11.0	1566	14.9
Seropositive with undetectable viral load									290	50.3	376	62.8	1183	75.5
≥ 1 episode of anal intercourse within the previous 12 months with any casual partner*														
All respondents	951	70.7	1914	81.4	1646	83.9	2007	83.9	3082	87.2	3570	88.9	6678	91.4
Untested	132	52.2	271	73.2	173	77.6	226	75.8	321	77.7	361	80.0	581	84.3
Seronegative	631	73.3	1242	80.7	1186	83.3	1462	83.4	2226	87.1	2587	88.8	4751	90.7
Seropositive	178	83.2	310	92.8	262	92.3	300	94.6	466	94.7	481	96.2	1332	97.5

*overall and according to self-reported HIV status.

Median age of respondents ranged from 30 years (Q1-Q2: 25–35) in 1995 to 35 years (26–44) in 2011. The highest educational category included 37.3% of the participants in 1991 and 47.3% in 2011 (*P*<0.0001). A significantly higher proportion of respondents lived outside the Paris region in 2004 and 2011—when questionnaires were available by internet—than in 1991–2000 (66.8% vs. 55.0%, *P*<0.0001).

From 1991 to 1995, the proportion of untested respondents fell significantly from 29.2% to 11.0% (*P*<0.0001), respectively and remained stable thereafter. The percentage of seropositive MSM fluctuated between surveys, with a low of 11.0% in 2004 and a high of 14.9% in 2011. The percentage of seropositive respondents reporting undetectable viral loads in their most recent blood sample rose from 50.3% in 2000 to 75.5% in 2011 (*P*<0.0001).

Anal intercourse with casual partners in the previous 12 months rose significantly in each serostatus group, and overall from 70.7% in 1991 to 91.4% in 2011 (*P*<0.0001). Over this entire period, seropositive respondents reported practising anal intercourse more often than seronegative and untested MSM (for example, in 2011: 97.5% vs. 90.7% vs. 84.3%, respectively, *P*<0.0001).

### Trends in UAId from survey to survey

**Fig 1:**
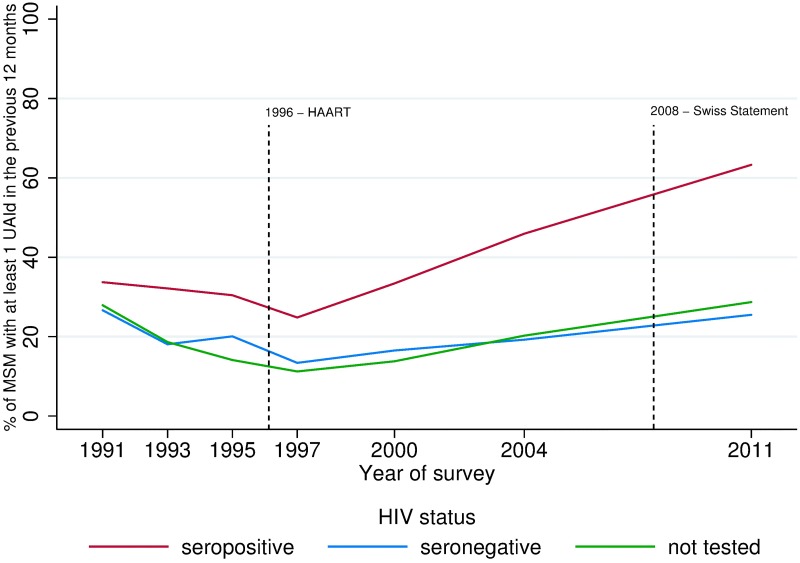
Trends in the percentage of respondents reporting at least one episode of UAId, as a function of HIV serostatus, in the Gay Press surveys from 1991 to 2011. UAId: unprotected anal intercourse in the 12 months before the survey with a casual partner of unknown or different HIV serostatus.

Globally, the percentage of men reporting UAId fell between 1991 and 1997. It then rose continuously from 1997 to 2011, irrespective of serostatus (Fig 1). Seropositive participants reported higher rates of UAId than their seronegative and untested counterparts throughout the period (see S1 Table for numerical values). The increase after 1997 was greater among seropositive respondents (*P* for interaction<0.0001). UAId rates did not differ significantly according to viral load detectability, even when we restricted the analysis of seropositive respondents to those who tested seropositive more than 2 years earlier (indicating that the practices reported during the previous year were those of someone aware of his seropositive status).

### Generational analysis of UAId

**Fig 2:**
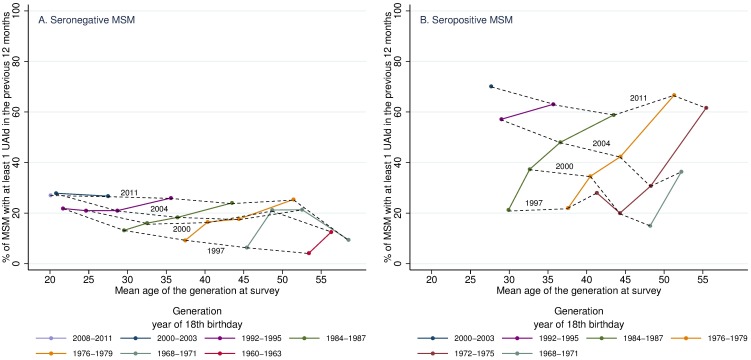
Longitudinal changes from 1997 to 2011 in the percentage of respondents who reported at least one episode of UAId. (A) seronegative respondents; (B) seropositive respondents. The dotted curves link the points obtained during the same survey year. UAId: unprotected anal intercourse in the 12 months before the survey with a casual partner of unknown or different HIV serostatus. Looking at Fig 2 we see that the generation of HIV seronegative MSM who turned 18 between 1984 and 1987 is represented by the green curve in Fig 2A. When responding to the 1997 Gay Press survey this generation's mean age was 29.6 years, and 13.2% reported having UAId at least once during the previous year. When this generation responded to the 2000 survey, the mean age was 32.5 years, and 16.0% reported having UAId at least once during the previous year. In 2004 and 2011, with mean ages of 36.5 years and 43.5 years, respectively, 18.3% and 24.0% of the seronegative MSM from this generation who responded to the surveys, reported having UAId at least once during the preceding year. Similarly, in Fig 2B, the green curve represents the generation of seropositive MSM that turned 18 years old between 1984 and 1987. This generation's mean age when they responded to the 1997 Gay Press survey was 29.9 years, and 21.3% reported having UAId at least once during the previous year. This generation’s mean age when they responded to the 2000 survey was 32.7 years, and 37.3% reported having UAId at least once during the previous year. In 2004 and 2011, with mean ages of 36.6 years and 43.5 years, respectively, 48.0% and 58.8% of the seropositive MSM this generation reported at least one episode of UAId during the preceding year.

The longitudinal graphs (Fig 2A and 2B) for the 1997 to 2011 period show that UAId increased with age, or in other terms, with time, for all generations. The increase was observed in both seronegative (Fig 2A) and seropositive MSM (Fig 2B), albeit at different speeds. The generation of seronegative MSM who turned 18 between 1984 and 1987 (green curve): in 1997 (mean age was 29.6 years) 13.2% reported at least one episode of UAId; this figure was 16.0% in 2000 (32.5 years), 18.3% in 2004 (36.5 years) and 24.0% in 2011 (43.5 years) (see S2 Table for complete numerical values).

Much higher UAId rates were reported in 2011 for all generations of seropositive respondents. Looking at the same example of the generation turning 18 between 1984 and 1987 (green curve, Fig 2B), we see that 21.3% reported UAId in 1997 (mean age of 29.9 years), 37.3% in 2000 (32.7 years), 48.0% in 2004 (36.6 years) and 58.8% in 2011 (43.5 years).

For any given age, and regardless of serostatus, the most recent generation always reported the highest percentage of UAId, except for the very youngest generation of seronegative MSM (those turning 18 years old in 2008–2011): their UAId rate was not higher than that of the 2000–2003 generation (i.e. turning 18 in 2000–2003) at the same age (27.0% vs. 27.9%, *P* = 0.86).

In the 2011 survey, in seropositive and seronegative MSM, the percentage of respondents reporting UAId was similar irrespective of age (dotted curves) for respondents up to 50 years old. This finding was not observed in any of the preceding surveys and is most likely associated with the major trends over time that affected all generations simultaneously. Among seronegative respondents, this increase was particularly notable among the oldest generations (i.e., those who turned 18 years old before 1976).

## Discussion

The Gay Press surveys, beginning in 1985, are one of the rare epidemiological tools in the world able to retrace the trajectory of prevention practices in MSM right from the onset of the HIV epidemic.

During the 1990s, the percentages of seropositive and seronegative MSM reporting UAId fell to relatively low and similar values. The increase after 1997, however, was more marked among seropositive MSM. Irrespective of HIV serostatus, the decrease in UAId between 1991 and 1997 and its subsequent rise were observed in all generations of respondents. This corresponds to a strong period effect. This result highlights how epidemiological and prevention contexts have shaped prevention practices among MSM, regardless of their age and their generation.

Our study has limitations. First, the non-probabilistic nature of the Gay Press surveys may limit the representativeness of the samples. In particular, it is likely that participation was linked to age [30,31], and associated with being sexually active or not. Accordingly, young participants (18–24 years) in the surveys would have been mainly composed of MSM who had an earlier sexual initiation, as shown in our previous paper [29]. Second, although we were not able to follow respondents at the individual level—because of the anonymous and voluntary nature of participation—the large-scale nationwide MSM surveys we implemented enabled us to create a generational longitudinal analysis, which in turn provided us with data about trends at the population level [32,33].

### Serostatus: An increasingly determinant factor in condomless anal intercourse

In 2011, 63.3% of seropositive respondents reported UAId with casual partners. Although the frequency of anal intercourse increased in the 1990s following the promotion of condom use [29], an increase in UAId was observed from 2000 onwards, following the widespread dissemination of highly active ARV combination therapies. During that period, UAId reached and exceeded levels from the early 1990s. At the individual level, our data show that from 2004 onwards, condomless sex was reported as frequently by seropositive MSM with a detectable viral load as by those with an undetectable viral load. This result is in line with those reported for the whole era of combined antiretroviral treatment in France [34] and in other high-resource countries [35–39]. They advocate for early antiretroviral ARV therapy and continue to promote the treatment-as-prevention concept, with the goal of reaching an undetectable viral charge as soon as possible.

In seronegative MSM, UAId increased from 1997 onward, with a return to the early 1990s level in 2011. This means however that the majority of seronegative MSM (three quarters in 2011) still continued to use condoms systematically. As shown elsewhere, among the respondents to the French Gay Press surveys [27,28], some seronegative MSM who did not use condoms systematically, engaged in seroadaptive practices such as seropositioning and serosorting, but at a lower rate than MSM in other countries [7,40]. Globally, these alternative strategies to condom use are not being adopted sufficiently to reverse the epidemic curve [8]. Indeed, these seroadaptive practices are only effective against HIV transmission if both partners have up-to-date knowledge about their serostatus [9].

Irrespective of MSM HIV status, alternative preventive tools, including biomedical prevention strategies, must be offered as part of a combination prevention programme that leaves individuals free to choose, without lessening the importance of the role which condoms play in prevention.

### Longitudinal analysis of preventive trajectories: Practices that evolved at the same time across all generations

The analyses of this corpus of surveys have shown that, just as for the trajectories of sexual practices [29], prevention practice trajectories in MSM have, on the whole, followed the same trends since 1991 in every generation. This is despite the fact that each individual has his own experience of the epidemic, especially in terms of the context of the epidemic at the time when he began his sexual life. From our perspective, 15 to 25 years after the onset of the epidemic, those who experienced the darkest years do not appear to have prevention behaviours which are different from those of later generations. Similarly, non-systematic condom use is not limited to younger generations. Certainly, MSM aged 20–24 years in 2011 reported more UAId than those aged 20–24 years in 1997, but this was true for all generations when comparing 2011 to 1997. Moreover, in 2011 the level of UAId in seronegative MSM was very similar for all the generations. This is despite the fact that pre-2011 surveys indicated different levels of UAId according to generation. We see here the value of generational analyses, which enable us to understand the dynamics that lead to a specific situation at a given time *t*.

The symmetry of the trends observed in our study confirms that the socio-epidemiological context has a strong influence on the sexuality of all MSM, predominating over historical generational specificities. In other words, the homogeneity observed in the trends reveals a period effect that has continued to shape the prevention behaviours of the entire MSM population throughout their lives.

It also highlights gay community preventive norms that evolved across ages and generations and which have taken place in a context of profound social transformations in different fields, as previously described [29]. More specifically, the last two decades in France have been marked by important legislative changes with regard to sexual minorities, such as the civil union (civil solidarity pact, PaCS) in 1999 [41], and then same sex marriage in 2013 [42]. These two developments have contributed to greater visibility of homosexuals in the public space and have been accompanied by increased social acceptance of homosexuality [43]. Moreover, at the end of the 1990s, controversy surrounding barebacking divided the community authorities on questions of prevention [44]. At the same time, the emergence of new ways to meet partners virtually, such as internet dating sites [45] and smartphone-based applications [46], have reconfigured spaces of sexual socialization to the detriment of traditional meeting places [47].

While these social evolutions have generally affected all MSM, they have preceded or have been concomitant with the entry into sexuality of the youngest MSM. Given that some studies have shown the influence of the context of entry into sexuality on sexual trajectories in the general population [48], it would seem particularly important to follow these young generations who have less uniform preventive practices than previous generations of the same age. More generally, a closer look at the social context in the evaluation and implementation of new prevention strategies represents, for some authors, an important field of study to develop in order to improve the effectiveness of health actions [49].

## Conclusion

The trends seen in every generation studied show that prevention campaigns and tools must target MSM of all ages, and at the same time, must leave the way open for specific approaches adapted to different age groups and different populations. Our results tend to suggest that new biomedical tools, such as PrEP, will soon be widely disseminated among those MSM most exposed to HIV risk. This fact underlines the major stakes involved for all MSM in terms of communicating, understanding, and taking ownership of the new preventive strategies.

## Supporting information

S1 Table Numerical values corresponding to Fig 1: Trends in the percentage of respondents reporting at least one episode of UAId. (XLSX)

S2 Table Numerical values corresponding to Fig 2: Longitudinal changes from 1997 to 2011 in the percentage of respondents who reported at least 1 episode of UAId. (XLSX)

## References

[1] Beyrer C, Sullivan P, Sanchez J, Baral SD, Collins C, Wirtz AL, et al The increase in global HIV epidemics in MSM. AIDS Lond Engl. 2013 11 13;27(17):2665–78. 10.1097/01.aids.0000432449.30239.fe 23842129

[2] Berg RC, Ross MW, Weatherburn P, Schmidt AJ. Structural and environmental factors are associated with internalised homonegativity in men who have sex with men: findings from the European MSM Internet Survey (EMIS) in 38 countries. Soc Sci Med 1982. 2013 2;78:61–9. 10.1016/j.socscimed.2012.11.033 23261257

[3] Sullivan PS, Jones JS, Baral SD. The global north: HIV epidemiology in high-income countries. Curr Opin HIV AIDS. 2014 3;9(2):199–205. 10.1097/COH.0000000000000039 24445370

[4] Schiltz M. Les homosexuels face au sida : enquête 1995 Regards sur une décennie d’enquêtes. CAMS CERMES ANRS; 1998.

[5] Velter A, Bouyssou-Michel A, Jauffret-Roustide M, de Busscher P., Semaille C. Rapport Enquête Presse Gay 2004. Saint-Maurice: InVS ANRS; 2007.

[6] Chen Y-H, Snowden JM, McFarland W, Raymond HF. Pre-exposure Prophylaxis (PrEP) Use, Seroadaptation, and Sexual Behavior Among Men Who Have Sex with Men, San Francisco, 2004–2014. AIDS Behav. 2016 3 16; 10.1007/s10461-016-1357-2 26983951

[7] Paz-Bailey G, Mendoza M, Finlayson T, Wejnert C, Le B, Rose C, et al Trends in condom use among men who have sex with men in the united states: the role of antiretroviral therapy and sero-adaptive strategies. AIDS Lond Engl. 2016 5 5; 10.1097/QAD.0000000000001139 PMC5838316 27149088

[8] Phillips AN, Cambiano V, Nakagawa F, Brown AE, Lampe F, Rodger A, et al Increased HIV incidence in men who have sex with men despite high levels of ART-induced viral suppression: analysis of an extensively documented epidemic. PloS One. 2013;8(2):e55312 10.1371/journal.pone.0055312 23457467 PMC3574102

[9] van den Boom W, Konings R, Davidovich U, Sandfort T, Prins M, Stolte IG. Is serosorting effective in reducing the risk of HIV infection among men who have sex with men with casual sex partners? J Acquir Immune Defic Syndr 1999. 2014 3 1;65(3):375–9. 10.1097/QAI.0000000000000051 PMC3947546 24189150

[10] McDaid LM, Hart GJ. Serosorting and strategic positioning during unprotected anal intercourse: are risk reduction strategies being employed by gay and bisexual men in Scotland? Sex Transm Dis. 2012 9;39(9):735–8. 10.1097/OLQ.0b013e31825a3a3c 22902673

[11] Philip SS, Yu X, Donnell D, Vittinghoff E, Buchbinder S. Serosorting is associated with a decreased risk of HIV seroconversion in the EXPLORE Study Cohort. PloS One. 2010;5(9). 10.1371/journal.pone.0012662 PMC2936578 20844744

[12] Vallabhaneni S, Li X, Vittinghoff E, Donnell D, Pilcher CD, Buchbinder SP. Seroadaptive practices: association with HIV acquisition among HIV-negative men who have sex with men. PloS One. 2012;7(10):e45718 10.1371/journal.pone.0045718 23056215 PMC3463589

[13] Stromdahl S, Hickson F, Pharris A, Sabido M, Baral S, Thorson A. A systematic review of evidence to inform HIV prevention interventions among men who have sex with men in Europe. Euro Surveill Bull Eur Sur Mal Transm Eur Commun Dis Bull. 2015;20(15). 10.2807/1560-7917.es2015.20.15.21096 25953133

[14] Grant RM, Lama JR, Anderson PL, McMahan V, Liu AY, Vargas L, et al Preexposure chemoprophylaxis for HIV prevention in men who have sex with men. N Engl J Med. 2010 12 30;363(27):2587–99. 10.1056/NEJMoa1011205 21091279 PMC3079639

[15] Molina J-M, Capitant C, Spire B, Pialoux G, Cotte L, Charreau I, et al On-Demand Preexposure Prophylaxis in Men at High Risk for HIV-1 Infection. N Engl J Med. 2015 12 3;373(23):2237–46. 10.1056/NEJMoa1506273 26624850

[16] Nguyen V-K, Bajos N, Dubois-Arber F, O’Malley J, Pirkle CM. Remedicalizing an epidemic: from HIV treatment as prevention to HIV treatment is prevention. AIDS Lond Engl. 2011 1 28;25(3):291–3. 10.1097/QAD.0b013e3283402c3e 20962615

[17] Janiec J, Haar K, Spiteri G, Likatavicius G, Van de Laar M, Amato-Gauci AJ. Surveillance of human immunodeficiency virus suggests that younger men who have sex with men are at higher risk of infection, European Union, 2003 to 2012. Euro Surveill Bull Eur Sur Mal Transm Eur Commun Dis Bull. 2013;18(48):20644. 10.2807/1560-7917.es2013.18.48.20644 24308979

[18] Halkitis PN, Kapadia F, Siconolfi DE, Moeller RW, Figueroa RP, Barton SC, et al Individual, psychosocial, and social correlates of unprotected anal intercourse in a new generation of young men who have sex with men in New York City. Am J Public Health. 2013 5;103(5):889–95. 10.2105/AJPH.2012.300963 23488487 PMC3660046

[19] Balaji AB, Bowles KE, Le BC, Paz-Bailey G, Oster AM, NHBS Study Group. High HIV incidence and prevalence and associated factors among young MSM, 2008. AIDS Lond Engl. 2013 1 14;27(2):269–78. 10.1097/QAD.0b013e32835ad489 PMC5098328 23079807

[20] Pharris A, Quinten C, Tavoschi L, Spiteri G, Amato-Gauci AJ, ECDC HIV/AIDS Surveillance Network. Trends in HIV surveillance data in the EU/EEA, 2005 to 2014: new HIV diagnoses still increasing in men who have sex with men. Euro Surveill Bull Eur Sur Mal Transm Eur Commun Dis Bull. 2015 11 26;20(47). 10.2807/1560-7917.ES.2015.20.47.30071 26625124

[21] Beltzer N, Saboni L, Sauvage C, Lydié N, Semaille C, Warszawski J, et al An 18-year follow-up of HIV knowledge, risk perception, and practices in young adults. AIDS Lond Engl. 2013 3 27;27(6):1011–9. 10.1097/QAD.0b013e32835e1583 23698065

[22] Cazein F, Pillonel J, Le Strat Y, Pinget R, Le Vu S, Brunet S, et al Découvertes de séropositivité VIH et de sida, France, 2003–2013. Bull Epidémiol Hebd. 2015;9–10:152–61.

[23] Lot F, Smati J, Montlahuc C, Cazein C, Barin F, Le Strat Y, et al Découvertes de séropositivité VIH chez les jeunes en France, 2003–2013. Bull Epidémiol Hebd. 2015;40–41:744–51.

[24] Le Vu S, Le Strat Y, Barin F, Pillonel J, Cazein F, Bousquet V, et al Population-based HIV-1 incidence in France, 2003–08: a modelling analysis. Lancet Infect Dis. 2010 10;10(10):682–7. 10.1016/S1473-3099(10)70167-5 20832367

[25] Infection par le VIH/SIDA et les IST. Point épidémiologique du 23 novembre 2015. / Actualités / Infection à VIH et sida / VIH-sida / IST / Maladies infectieuses / Dossiers thématiques / Accueil [Internet]. [cited 2016 Feb 26]. http://www.invs.sante.fr/Dossiers-thematiques/Maladies-infectieuses/VIH-sida-IST/Infection-a-VIH-et-sida/Actualites/Infection-par-le-VIH-SIDA-et-les-IST.-Point-epidemiologique-du-23-novembre-2015

[26] Adam P, Hauet E, Caron C. Recrudescence des prises de risque et des MST parmi les gays Résultats préliminaires de l’Enquête Presse Gay 2000. Saint-Maurice: InVS ANRS; 2011.

[27] Velter A, Bouyssou-Michel A, Arnaud A, Semaille C. Do men who have sex with men use serosorting with casual partners in France? Results of a nationwide survey (ANRS-EN17-Presse Gay 2004). Euro Surveill Bull Eur Sur Mal Transm Eur Commun Dis Bull. 2009;14(47). 10.2807/ese.14.47.19416-en 19941805

[28] Velter A, Saboni L, Sommen C, Bernillon P, Bajos N, Semaille C. Sexual and prevention practices in men who have sex with men in the era of combination HIV prevention: results from the Presse Gays et Lesbiennes survey, France, 2011. Euro Surveill Bull Eur Sur Mal Transm Eur Commun Dis Bull. 2015;20(14). 10.2807/1560-7917.es2015.20.14.21090 25884150

[29] Méthy N, Velter A, Semaille C, Bajos N. Sexual behaviours of homosexual and bisexual men in France: a generational approach. PloS One. 2015;10(3):e0123151 10.1371/journal.pone.0123151 25816322 PMC4376702

[30] Marcus U, Hickson F, Weatherburn P, Schmidt AJ, EMIS network. Age biases in a large HIV and sexual behaviour-related internet survey among MSM. BMC Public Health. 2013;13:826 10.1186/1471-2458-13-826 24020518 PMC3847490

[31] Kellogg TA, Hecht J, Bernstein K, McFarland W, Connors A, Perloff L, et al Comparison of HIV behavioral indicators among men who have sex with men across two survey methodologies, San Francisco, 2004 and 2008. Sex Transm Dis. 2013 9;40(9):689–94. 10.1097/01.olq.0000431354.96087.50 23945424

[32] Elford J, Jeannin A, Spencer B, Gervasoni JP, van de Laar MJ, Dubois-Arber F, et al HIV and STI behavioural surveillance among men who have sex with men in Europe. Euro Surveill Bull Eur Sur Mal Transm Eur Commun Dis Bull. 2009;14(47). 10.2807/ese.14.47.19414-en 19941807

[33] Paquette D, De Wit J. Sampling methods used in developed countries for behavioural surveillance among men who have sex with men. AIDS Behav. 2010 12;14(6):1252–64. 10.1007/s10461-010-9743-7 20614177

[34] Seng R, Rolland M, Beck-Wirth G, Souala F, Deveau C, Delfraissy J-F, et al Trends in unsafe sex and influence of viral load among patients followed since primary HIV infection, 2000–2009. AIDS Lond Engl. 2011 4 24;25(7):977–88. 10.1097/QAD.0b013e328345ef12 21358375

[35] Kennedy C, O’Reilly K, Medley A, Sweat M. The impact of HIV treatment on risk behaviour in developing countries: a systematic review. AIDS Care. 2007 7;19(6):707–20. 10.1080/09540120701203261 17573590

[36] Crepaz N, Marks G, Liau A, Mullins MM, Aupont LW, Marshall KJ, et al Prevalence of unprotected anal intercourse among HIV-diagnosed MSM in the United States: a meta-analysis. AIDS Lond Engl. 2009 8 24;23(13):1617–29. 10.1097/QAD.0b013e32832effae 19584704

[37] Venkatesh KK, Flanigan TP, Mayer KH. Is expanded HIV treatment preventing new infections? Impact of antiretroviral therapy on sexual risk behaviors in the developing world. AIDS Lond Engl. 2011 10 23;25(16):1939–49. 10.1097/QAD.0b013e32834b4ced PMC7295031 21811137

[38] Kaye DK, Kakaire O, Osinde MO, Lule JC, Kakande N. The impact of highly active antiretroviral therapy on high-risk behaviour of HIV-infected patients in sub-Saharan Africa. J Infect Dev Ctries. 2013 6;7(6):436–47. 10.3855/jidc.2644 23771287

[39] Chen Y. Treatment-related optimistic beliefs and risk of HIV transmission: a review of recent findings (2009–2012) in an era of treatment as prevention. Curr HIV/AIDS Rep. 2013 3;10(1):79–88. 10.1007/s11904-012-0144-6 23239272 PMC3567224

[40] Hart GJ, Elford J. Sexual risk behaviour of men who have sex with men: emerging patterns and new challenges. Curr Opin Infect Dis. 2010 2;23(1):39–44. 10.1097/QCO.0b013e328334feb1 19949328

[41] Broqua C. La crise de la normalisation : expérience et condition sociales de l’homosexualité en France In: Homosexualités au temps du sida Tensions sociales et identitaires. Broqua C, Lert F, Souteyrand Y. Paris: ANRS; 2003.

[42] Chauvin S, Lerch A. Sociologie de l’homosexualité. Paris: La Découverte; 2013.

[43] Bajos N, Beltzer N. Homo-/bisexuality: token acceptance and social and protective vulnerabilities In: Sexuality in France; Practices, Gender & Health. Bajos N, Bozon M. Oxford: The Bardwell Press; 2012.

[44] Girard G. HIV risk and sense of community: French gay male discourses on barebacking. Cult Health Sex. 2016 1;18(1):15–29. 10.1080/13691058.2015.1063813 26279071

[45] Bolding G, Davis M, Hart G, Sherr L, Elford J. Gay men who look for sex on the Internet: is there more HIV/STI risk with online partners? AIDS Lond Engl. 2005 6 10;19(9):961–8. 10.1097/01.aids.0000171411.84231.f6 15905678

[46] Hull P, Mao L, Prestage G, Zablotska I, de Wit J, Holt M. The use of mobile phone apps by Australian gay and bisexual men to meet sex partners: an analysis of sex-seeking repertoires and risks for HIV and STIs using behavioural surveillance data. Sex Transm Infect. 2016 4 19; 10.1136/sextrans-2015-052325 27095378

[47] Zablotska IB, Holt M, Prestage G. Changes in gay men’s participation in gay community life: implications for HIV surveillance and research. AIDS Behav. 2012 4;16(3):669–75. 10.1007/s10461-011-9919-9 21424273

[48] Bozon M. First intercourse and first relationship: long anticipated trasitions In: Sexuality in France; Practices, Gender & Health. Bajos N, Bozon M. Oxford: The Bardwell Press; 2012.

[49] Shoveller J, Viehbeck S, Ruggiero ED, Greyson D, Thomson K, Knight R. A critical examination of representations of context within research on population health interventions. Crit Public Health. 2016 10 19;26(5):487–500.

